# Nature Connection to Improve Staff Wellbeing at a Hampshire and Isle of Wight Healthcare NHS Foundation Trust Site in the New Forest National Park

**DOI:** 10.1192/bjo.2025.10426

**Published:** 2025-06-20

**Authors:** Laura Pridmore, Jennifer Wyllie

**Affiliations:** 1 New Forest National Park Authority, Lymington, United Kingdom; 2 Hampshire County Council, Winchester, United Kingdom; 3 Hampshire and Isle of Wight Healthcare NHS Foundation Trust, Tatchbury, United Kingdom

## Abstract

**Aims:** Tatchbury Mount is a large hospital site at the edge of the New Forest National Park (NFNP), home to four secure inpatient units, community teams, an 80-bed nursing home and management offices. It has an abundance of wooded areas and nature. The site is part of the NHS Forest scheme which aims to support the NHS in becoming more sustainable and to reach net zero carbon by 2040. Despite the natural attributes of the site the grounds are underused by staff and patients with the only sensory trail on the site overgrown and neglected.

The aims of our project are threefold: to firstly regenerate the sensory trail, encouraging staff to use their one-day allowance of volunteer leave alongside volunteers from the New Forest National Park Authority (hosting our fellowship). Secondly, to communicate to staff through the wellbeing team and champions network as well as engagement with clinical teams on site the benefits of spending time in nature and to increase awareness of the sensory trail. Finally, to better understand the barriers for NHS staff accessing nature at the site.

**Methods:** We have liaised with key stakeholders in regeneration of the sensory trail including Hampshire County Council Public health wellbeing team, estates department, NFNPA volunteers and the trust wellbeing team. Two days have been agreed to regenerate the site with advertising created as well as communications through the wellbeing champion network. We will continue to gather insights from staff and have prepared a questionnaire to be completed by NHS staff to evaluate the impact of volunteering on wellbeing.

**Results:** Through this project we have made links with the Wellbeing team for Hampshire and IoW trust and Hampshire County Council PH wellbeing team. We have identified that staff including the trust wellbeing team are currently not aware of the sensory trail and by using the COM-B model of behaviour change we have identified that the barriers to access nature are multifactorial and link with each other. These include time/workload, staff shortages, culture not to take a break, knowledge that the trails exist and physical health/injuries.

**Conclusion:** There is great opportunity for NHS sites across the country to be used by staff to connect with nature and in turn enhance wellbeing.

The involvement of the trust Wellbeing team, wellbeing champions and management is vital to ensure that the embedded culture surrounding taking breaks and focusing on wellbeing is recommended and supported.

